# Strategies for Diagnosing and Treating Disseminated Methicillin-Sensitive Staphylococcus aureus Infections: Insights From a Pustular Eruption

**DOI:** 10.7759/cureus.67516

**Published:** 2024-08-22

**Authors:** Kritin K Verma, Ethan J Matthew, Ryan Wealther, Rohan Pendse, Michelle Tarbox

**Affiliations:** 1 School of Medicine, Texas Tech University Health Sciences Center, Lubbock, USA; 2 Dermatology, Texas Tech University Health Sciences Center, Lubbock, USA; 3 Neurosurgery, Texas Tech University Health Sciences Center, Lubbock, USA

**Keywords:** immunocompromised patients, disseminated infection, vesiculopustular eruption, clinicopathologic correlation, disseminated staphylococcus aureus infection, staphylococcus aureus

## Abstract

A 63-year-old immunocompromised male with a history of renal transplant and stage III large B-cell non-Hodgkin lymphoma undergoing rituximab, cyclophosphamide, doxorubicin, vincristine, and prednisone (R-CHOP) therapy presented with fever and a disseminated pustular eruption. Initial laboratory values indicated septicemia. Differential diagnoses included Sweet’s syndrome, septic emboli, and leukocytoclastic vasculitis. Punch biopsies and bacterial cultures confirmed disseminated methicillin-sensitive *Staphylococcus aureus* (MSSA) infection. Histopathology revealed intraepidermal vesiculopustules and bacterial cocci colonies in the superficial dermis, suggesting hematogenous spread. The patient’s indwelling venous access port was identified as the infection source and removed. Treatment included antibiotics such as cefepime, vancomycin, fluconazole, and acyclovir, as well as filgrastim for neutropenia. Following port removal and a four-week course of ceftriaxone, the patient’s condition improved. This case highlights the importance of clinicopathologic correlation in diagnosing and managing disseminated staphylococcal infections in immunocompromised patients. The rare presentation of vesiculopustular eruptions secondary to MSSA emphasizes the need for prompt identification and treatment to prevent severe complications. This report contributes to the limited literature on disseminated staphylococcal infections presenting as vesiculopustular eruptions in immunocompromised individuals.

## Introduction

*Staphylococcus aureus* is a component of normal skin flora and a common pathogen responsible for various clinical infections, including skin and soft tissue infections, endocarditis, osteomyelitis, and bacteremia [1]. Pathogenic strains of *Staphylococcus aureus *colonize human skin and mucous membranes, most commonly the anterior nares, making eradication difficult. Colonization may lead to recurrent and life-threatening infections and sequelae such as impetigo, necrotizing fasciitis, staphylococcal scalded skin syndrome, and disseminated infection [1-3].

This report presents the case of a patient with disseminated vesiculopustular eruptions on various parts of the body. The patient was diagnosed with a disseminated methicillin-sensitive *Staphylococcus aureus* (MSSA) infection, which was treated with antibiotics and the removal of the venous access port.

## Case presentation

**Figure 1:**
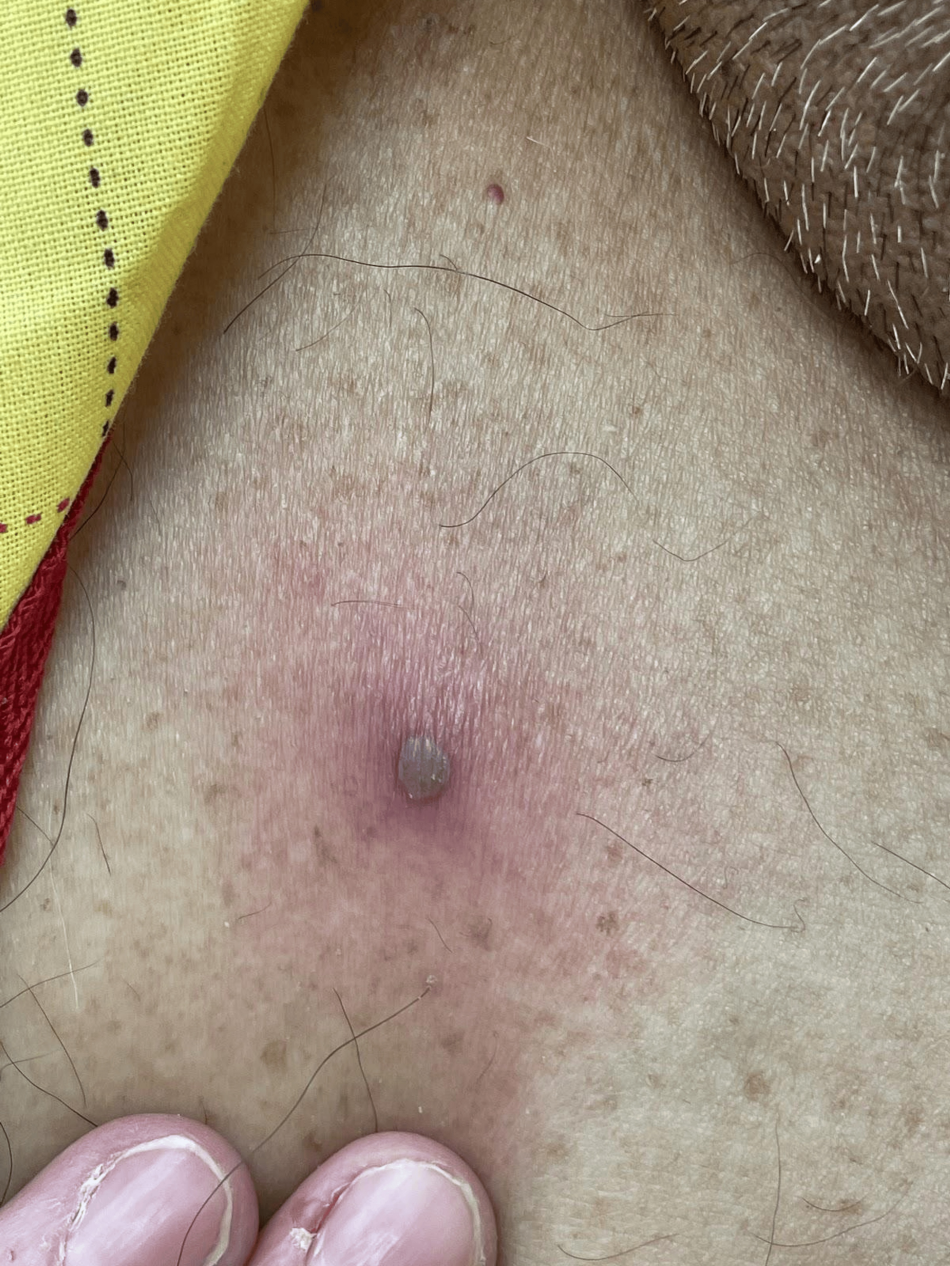
Purpuric and painful vesicle from Staphylococcus aureus infection. Close-up of a vesicle in which the patient complained of pain and moderate pruritus with the lesions.

A 63-year-old male with a history of renal transplant and currently being treated with rituximab, cyclophosphamide, doxorubicin, vincristine, and prednisone (R-CHOP) therapy regimen for stage III large B-cell non-Hodgkin lymphoma presented to the emergency department. He complained of a three-day history of fever and a worsening pustular eruption (Figure 1).

**Figure 2:**
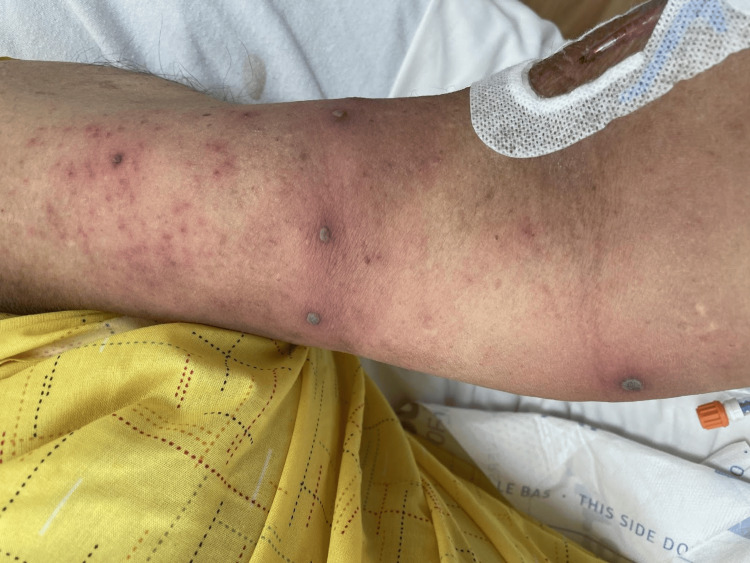
Painful and itchy purpuric vesicles and pustules on the extremities. Disseminated pustule eruption was present on the chest, axilla, shoulders, left and right arms, hands, bilateral lower extremities, face, abdomen, back, and feet.

Physical examination revealed a disseminated pustular eruption present on the trunk, bilateral upper and lower extremities, face, and feet (Figure 2).

Pain and moderate pruritus accompanied the onset of the lesions. The patient was admitted to the hospital and treated with cefepime, vancomycin, fluconazole, and acyclovir, and two doses of filgrastim were given for neutropenia secondary to septicemia, which was diagnosed by the obtained laboratory values (Table 1).

**Table 1:** Initial laboratory values indicative of septicemia.

Parameter	Result	Reference range
White blood cells	1.37 × 10³ cells/mm³	4.0–11.0 × 10³ cells/mm³
Procalcitonin	2.47 ng/mL	<0.1 ng/mL
Sedimentation rate	71 mm/hour	0–20 mm/hour
C-reactive protein	28.6 mg/L	<3.0 mg/L

**Figure 3:**
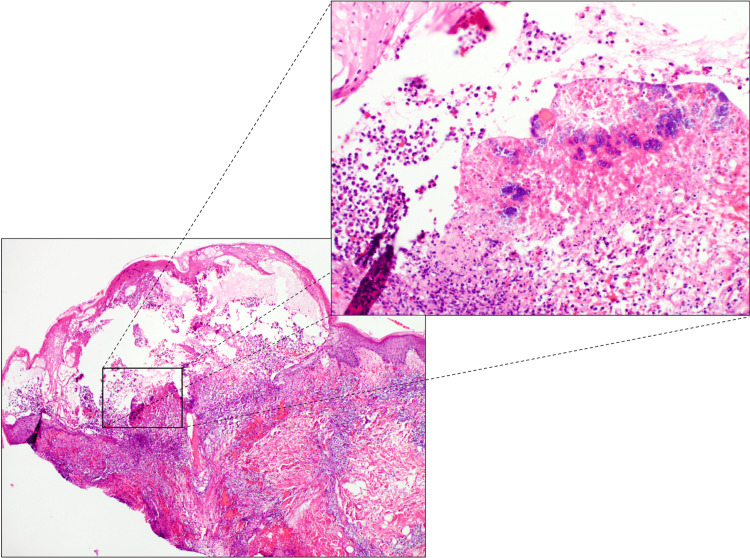
H&E findings taken from a punch biopsy without (bottom left) (2×) and with (top right) (10×) magnification. Histological sections display an attenuated epidermis overlying a vesiculopustule. There are numerous large colonies of bacterial cocci within the superficial dermis, and there is a brisk mixed inflammatory infiltrate within the superficial dermis extending to the deep dermis surrounding the dermal vasculature. H&E: hematoxylin and eosin

On day two of admission, his fever improved, but the pustular eruption persisted. Initial clinical differential diagnoses included septic emboli, leukocytoclastic vasculitis, Sweet’s syndrome, septic vasculitis, herpes infection, and gonococcemia. Punch biopsies were performed on the lesions on the chest and left arm, and a bacterial culture of the lesion from the left arm was performed, which later grew MSSA. Hematoxylin and eosin stain of the punch biopsy on the chest showed an attenuated epidermis overlying an intraepidermal vesiculopustule with numerous large colonies of bacterial cocci within the superficial dermis. A mixed perivascular inflammatory infiltrate was present in the superficial dermis (Figure 3). No other special stain was performed.

Initial workup of the neutropenic fever yielded negative blood cultures, and echocardiography did not demonstrate valvular pathology. However, the dermatopathology findings were highly suggestive of a hematogenous origin for the observed bacterial cocci in the tissue. These findings and the positive culture date of the wounded prompted the removal of his indwelling venous access port, which was subsequently found to be culture-positive for pan-sensitive MSSA. After starting the patient on a four-week course of ceftriaxone and removing the port, the pustular lesions began to regress, and the patient was sent home in stable condition.

## Discussion

*Staphylococcus aureus* is a common pathogen responsible for various common clinical infections [1-3]. In this case, the patient’s underlying bacteremia of MSSA presented as septic emboli in the form of a disseminated pustular eruption present on the trunk, bilateral upper and lower extremities, face, and feet. Disseminated staphylococcal infections are not uncommon, especially in children, and are often accompanied by characteristic systemic symptoms such as staphylococcal pneumonia, osteomyelitis, or cardiac involvement [4]. Cases of staphylococcal sepsis in children are associated with pulmonary infections, and skin findings are proposed to indicate a higher mortality rate [4]. Another case of a 12-year-old patient reported disseminated staphylococcal septicemia with the presentation of pustular lesions on his face and trunk [4]. Clinical presentation of disseminated staphylococcal septicemia presenting as a vesiculopustular eruption is rarely cited in the literature [1,4]. In this case, the presentation of a vesiculopustular eruption secondary to an infected central venous catheter serving as a nidus of infection may be a novel presentation in this immunocompromised patient.

Bacterial cultures of the lesions of the left arm and central venous access port grew MSSA, suggesting an initial infection site at the central venous access port, with later presumptive disseminated septic emboli culminating in his presentation of vesiculopustular lesions on the trunk, bilateral upper and lower extremities, face, and feet [1]. Histopathological evidence of an attenuated epidermis overlying a pustule with large colonies of bacterial cocci within the superficial dermis raised significant suspicion of hematological spread, prompting the removal of the central venous access port by the primary team. MSSA infections are often treated with penicillinase-resistant penicillins and cephalosporins. The patient improved on a course of nafcillin while in the hospital and was successfully discharged home with a four-week course of ceftriaxone with the resolution of vesicular pustular [5].

Staphylococcal bacteremia complications can be severe, including endocarditis, osteomyelitis, abscess development, pneumonia, mediastinitis, pericarditis, and septic shock [6,7]. To avoid significant complications, staphylococcal bacteremia must be treated as soon as possible [1,6,7]. While antistaphylococcal penicillins such as nafcillin are often the first-line treatment for MSSA bacteremia, alternatives may be considered based on culture sensitivities [1]. Additionally, as in this case, immunosuppressed patients are more likely to experience severe infections from staphylococcal bacteremia and may present with symptoms of septic embolization [8].

This report describes a rare cutaneous presentation of disseminated staphylococcal infection and its treatment regime [1,3]. The differential diagnosis of vesiculopustular eruptions is broad and includes disseminated gonococci, secondary syphilis, disseminated bacterial infection, Sweet’s syndrome, and disseminated herpes simplex virus infection [1]. Non-infectious entities, including pustular psoriasis, acute generalized exanthematous pustulosis, and eosinophilic folliculitis, could also be considered. Histopathology of these conditions will show superficially seated pustules with polymorphic neutrophils with or without eosinophils, which would histologically distinguish these entities from the findings of the infectious entities. In this case, the histologic findings were the key to guiding and narrowing down the differential diagnosis to infectious entities causing disseminated vesiculopustular eruptions and ensuring that the patient received the correct treatment course. The case report emphasizes the importance of clinicopathologic correlation in guiding the diagnosis algorithm for hospitalized patients with generalized vesiculopustular eruptions, especially those with immunosuppression and suspected infections.

## Conclusions

This case report underscores the importance of clinicopathologic correlation in diagnosing and managing disseminated staphylococcal infections in immunocompromised patients. The rare presentation of vesiculopustular eruptions secondary to MSSA infection highlights the need for clinicians to consider atypical manifestations of common pathogens in this patient population. The successful management of this patient through antibiotic therapy and the removal of the infected port emphasizes the critical role of source control in treating disseminated infections. This report adds to the limited literature on disseminated staphylococcal infections presenting as vesiculopustular eruptions and serves as a valuable reference for clinicians managing similar cases in immunocompromised individuals.
